# Towards control strategies for microplastics in urban water

**DOI:** 10.1007/s11356-020-10064-z

**Published:** 2020-07-14

**Authors:** Emma Fältström, Stefan Anderberg

**Affiliations:** 1 Environmental Technology and Management, Department of Management and Engineering, Linköping University, SE-581 83 Linköping, Sweden; 2 Sweden Water Research AB, Ideon Science Park, Scheelevägen 15, SE-223 70 Lund, Sweden

**Keywords:** Control strategies, Pollution management, Stormwater, Substance flow analysis, SFA, Wastewater, Urban water

## Abstract

Microplastics (plastic particles < 5 mm) is a pollution of growing concern. Microplastic pollution is a complex issue that requires systematic attempts to provide an overview and avoid management solutions that have marginal effects or only move the pollution problem. Substance flow analysis (SFA) has been proposed as a useful tool to receive such an overview and has been put forward as valuable for substance management. However, as the research on microplastics has only emerged recently, detailed and reliable SFAs are difficult to perform. In this study, we use three SFA studies for three pollutants (cadmium, copper and pharmaceuticals) to compare flows and strategies to control the flows. This in order to seek guidance for microplastic management and evaluate potential strategies for controlling microplastics. The analysis shows that there has been rigorous control on different levels to abate pollution from cadmium, copper and pharmaceuticals, but where in the system the major control measures have been carried out differ. For microplastics, there are many potential solutions, both in terms of preventive actions and treatment depending on the type of source. When forming management plans for microplastics, the responsibility for each measure and the impact on the whole urban system should be taken into consideration as well as which receiving compartments are particularly valuable and should be avoided.

## Introduction

Environmental pollution from a wide range of sources is concentrated in urban areas (Holten Lützhøft et al. 2012), and urban waters, i.e. stormwater and wastewater, are important pathways (Revitt et al. 2013). Microplastics (plastic particles < 5 mm) is a new type of pollution that has received increased attention in recent years. The widespread presence of microplastics in the environment seems to have adverse effects on both marine (Cole et al. 2011; Wright et al. 2013) and freshwater biota (Eerkes-Medrano et al. 2015). The research on microplastics has grown rapidly and made important conceptual as well as empirical progress. However, microplastics research is still in its infancy, and research results are often uncertain, and sometimes contradictory. This makes it difficult to develop a sufficiently reliable overview of sources and pathways that can be used as a basis for comprehensive and efficient abatement strategies. Inspired by Sedlak (2017), who argues that there are lessons to be learned from past pollution issues when managing microplastics, this study has sought guidance for microplastic management from SFA studies of substances with a longer management history.

Microplastics can originate both from marine and land-based sources. Insufficient waste management (Jambeck et al. 2015), road traffic (Kole et al. 2017) and production spill (Karlsson et al. 2018) have been identified as significant land-based sources of microplastic pollution. Textile fibres from the washing of synthetic material (Browne et al. 2011) as well as microbeads in personal care products (PCPs) (Napper et al. 2015) are also considered important sources.

Microplastics are omnipresent in urban areas (Tibbetts et al. 2018) and found in both stormwater (Borg Olesen et al. 2019; Liu et al. 2019) and wastewater (Ngo et al. 2019). Wastewater treatment plants (WWTPs) show a high capacity to retain microplastics, often up to 99%, even without any advanced treatment (Carr et al. 2016; Lares et al. 2018; Murphy et al. 2016; Simon et al. 2018). Despite this, elevated levels have been reported in the recipient waters of WWTPs (Estahbanati and Fahrenfeld 2016).

The time-consuming and expensive analysis of microplastics, as well as the lack of standardised methods (Hidalgo-Ruz et al. 2012; Li et al. 2018), makes it difficult to acquire sufficient information for a detailed quantitative overview of flows of microplastics. Despite this, attempts to estimate the sources and pathways of microplastic pollution have been made on different levels. For example, the major sources have been estimated for Sweden (Magnusson et al. 2016), Denmark (Lassen et al. 2015), Norway (Sundt et al. 2014) and Germany (Essel et al. 2015), as well as in the Swedish capital Stockholm (Ejhed et al. 2018). These estimates are, however, subject to large uncertainties. First attempts to model microplastics flows for larger regions have also been made (Siegfried et al. 2017).

The scarce and uncertain knowledge and lacking overview make it difficult to develop strategies for reducing microplastic pollution (Pettipas et al. 2016). Still, there is a need to manage microplastics and there have been many proposed measures and a few have been implemented, but the measures have not been evaluated from broader perspectives. To avoid single initiatives with limited effects, it is important with an ambition to develop preliminary overviews that can form the basis for more comprehensive and efficient strategies. For developing such an overview, the analysis of microplastic pollution may benefit from a systematic approach. A systems perspective can help avoid solutions that move pollution from one part of the system to another or outside the urban area (Eriksson et al. 2011; Revitt et al. 2013). Substance flow analysis (SFA) has been proposed as useful to get an overview of flows of pollutants and for grasping complexity (Lindqvist 2002). SFA has been used for many substances, including nutrients such as nitrogen (Li et al. 2020), metals such as silver (Amneklev et al. 2014), chromium (Anderberg et al. 1989) and copper (Amneklev et al. 2016b) and other types of pollution such as chloroparaffins (Eriksson et al. 2012) and parabens (Eriksson et al. 2008) on different spatial levels ranging from a specific flow within a city (Amneklev et al. 2016a) to the European Union (Sundseth et al. 2012).

At this point, the research on microplastics is not at a stage where a detailed SFA can be provided. However, SFA studies have been performed for other types of urban pollution, and these can inform ways forward in relation to the microplastic issue and help avoid similar problems that the management has encountered in connection with other substances.

In this study, we seek guidance for microplastic management by addressing the urban flows and pollution of substances with a longer management history. For this analysis, we have selected three cases of different substances in European city regions where flows have been analysed by SFA studies. The analysis focuses on the flows of the pollutants within the urban area and the strategies used for controlling the substance in the case cities.

## Methodological approach

### Substance flow analysis

Substance flow analysis is a comprehensive systems approach for analysing stocks and flows of different elements and compounds on various spatial levels. SFA studies commonly consist of three phases: (1) definition of the system and the system components, (2) quantification of stocks and flows and (3) interpretation (van der Voet 2002). The first phase consists of defining the system in terms of the processes, stocks and flows, as well as space and time restrictions, and defining the substance or substance group that will be investigated. In the second phase, the stocks and flows of the substance within the system are quantified. The results from phase 2 are then interpreted. This often consists of an evaluation of the reliability of the quantification, but some studies make efforts to frame and communicate the results to policy makers (van der Voet 2002).

SFA has been put forward as a powerful tool for detecting depletion and accumulations, developing resource-related strategies as well as supporting decisions on management priorities (Brunner 2012). SFA has also been argued to be a suitable tool for urban water management (Chèvre et al. 2011). The comprehensive overview is often seen as a strength of SFA, but the overview in itself has also been criticised for being insufficient for environmental management (Lindqvist and Eklund 2002). In this study, we take advantage of the overview in the SFA studies and use this to derive information about the flows that are relevant for the urban water system. We complement the information about the flows with information about the strategies used to tackle the pollution.

### System boundaries and selection of substances and cases

**Fig. 1:**
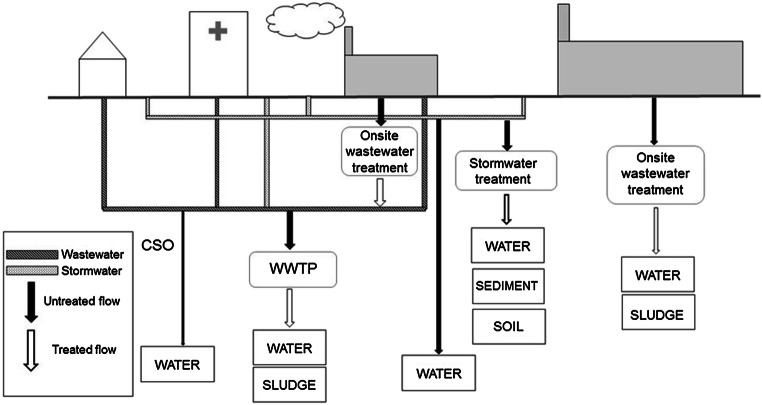
Conceptual illustration of the urban water system. CSO stands for combined sewer overflow and WWTP for wastewater treatment plant. Developed from Revitt et al. (2013)

Knowledge about the sources and pathways of microplastics is still limited, but urban water has been identified as an important pathway. Following the first step of the SFA methodology, defining the system, the system boundaries are set to the urban area and its water system, i.e. wastewater and stormwater (Fig. 1). Urban activities, as well as the infrastructure and buildings of the urban area, influence pollutant loads and pathways. Households, hospitals, and some enterprises all contribute to pollutant loads to the WWTP. Some enterprises have their own treatment before releasing the water to the wastewater system, while some large industries are not connected to the WWTP and rely only on their own treatment facilities. Stormwater often contains pollution from urban activities, such as road traffic and from urban structures, e.g. roofs (Sörme and Lagerkvist 2002). The stormwater can discharge to recipient waters directly or, via combined pipe systems, be treated at the WWTP. Combined sewer overflows (CSOs) are untreated or partially treated wastewater mixed with stormwater, which is released to the receiving water body (Chèvre et al. 2013). After Revitt et al. (2013) sludge, sediment, surface water and soil are seen as receiving compartments.

The choice of substances in this study was based on three criteria. Microplastics is considered an environmental problem, and therefore, the first criterion was that the selected substance has negative environmental consequences. Microplastics is a diverse pollutant group, why the second criterion was that the substances should differ in terms of their properties, their use and their history as environmental issues. The third criterion was that the pollutant, like microplastics, should be considered a problem in urban areas and in urban water. Based on these criteria, cadmium, copper and pharmaceuticals were selected. These substances have differences and similarities to microplastics and to each other. Pharmaceuticals, like microplastics, has rather recently been put forward as problematic, while cadmium and copper have been on the agenda longer. Further, pharmaceuticals consist of many different compounds, like microplastics, which can consist of many different polymers. Cadmium and copper are both naturally occurring elements, but while copper is considered an essential element, cadmium is not.

The search for SFA studies for the selected substances was limited to studies performed in urban areas in European countries because some similarity in contextual conditions in terms of governance, culture and economic development is desirable in relation to policy discussions. There are most often considerable uncertainties in the results of SFA studies and therefore not too much attention should be paid to the precise numbers (Amneklev et al. 2016b). Neither should the absolute numbers be regarded as most important in relation to the purpose of this paper; instead, it is the overview of flows provided by the SFA that is of primary importance. The selected SFA studies (Table 1) have all been performed at a city level and are all presented in peer-reviewed articles.

**Table 1:** SFAs included in this study

Substance	Reference	Location	Year
Cadmium (Cd)	Bergbäck et al. (2001)	Stockholm, Sweden	1995
Copper (Cu)	Kral et al. (2014)	Vienna, Austria	2008
Pharmaceuticals	Chèvre et al. (2013)	Lausanne, Switzerland	A particular year is not specified

### Analytical framework

For the comparative analysis, an analytical framework (Table 2), inspired by the SFA literature (particularly Eriksson et al. 2011; Lindqvist and Eklund 2002; Revitt et al. 2013; van der Voet et al. 1999) highlighting important flows in connection with the urban water system and related strategies for abatement, was used. The framework consists of two parts, flows and strategies. The aspects related to the flow part consist of the standard outputs of an SFA: major sources, pathways and receiving compartments. For keeping a reasonable overview, only the largest flows are included in the analysis. In connection with the strategy analysis, we first investigated if suggestions were made by the authors based on the results of their analysis. Then, we examined what abatement and control strategies were implemented. These strategies can be divided into three levels:

- Preventive (e.g. legislation or behavioural change)
- Decentralised treatment (at the source or in the urban area)
- Centralised treatment (at the WWTP)

**Table 2:** The framework developed in this study to assess the flows for the urban water system in the selected SFAs and the strategies used

Category	Aspect	Description/guiding question
Flows	Top sources	The two largest sources according to the SFA.
Flows	Top pathways	The two largest pathways according to the SFA, both before and after treatment
Flows	Top receiving compartments	The two largest receiving compartments for the pollution according to the SFA.
Strategies	Strategies suggested in SFA	Have the authors of the selected studies suggested any strategies?
Strategies	Strategies used	What strategies (preventive or treatment) have been implemented to abate the pollution?
Strategies	Status	What is the status of implementation of the strategies?
Strategies	Responsibility	On what level (EU, national or local) is the responsibility for implementing the strategies?
Strategies	Pollution movement	Do the strategies move the pollution from one compartment to another?

In addition, the responsibility for implementation of the strategies and measures was considered. Even if all the used SFA studies were on the city level, local authorities do not have full influence of the flows (Lindqvist and Eklund 2002). Still, practical implementation of policy and legislation can be a local responsibility. Three levels were considered: EU, national and local (i.e. municipal/city level). For microplastics, most of the strategies are not implemented yet. The responsibility for the strategies found were categorised based on the actors that most likely would have the responsibility if the strategy or measure were to be implemented.

A strength of SFA is that it can provide an overview of the system, which can be used for assessing the consequences of different actions and discover potential problems (Chèvre et al. 2011). One such problem concerns shifting the pollution (van der Voet et al. 1999). *Pollution movement* describes the potential problem that a strategy moves pollution from one compartment to another. It should be noted that pollution movement refers to the movement of the investigated pollutant and does not include if a strategy leads to increase in other types of pollution.

## Results and discussion

The largest sources, pathways and receiving compartments for the urban water system identified in the SFA studies for cadmium, copper and pharmaceuticals are presented in Table 3. The table summarises the abatement and control strategies, their status of implementation, which actors are responsible for implementation and if there has been a pollution movement to other compartments as a consequence of the implemented strategies. The three case SFAs are compared in two ways. First, similarities and differences in terms of largest sources, pathways and receiving compartments are discussed. Second, the strategies used for the different substances and cases are compared, and the strategies for each substance are compared to the flows of this substance. The last part of this section focuses on the development of strategies for microplastics and addresses opportunities and challenges with regards to the future management of microplastic pollution.

**Table 3:** Summary of the largest sources, pathways and receiving compartments for the urban water system according to the SFAs on cadmium (Bergbäck et al. 2001), copper (Kral et al. 2014) and pharmaceuticals (Chèvre et al. 2013) and the strategies used

Flows					Strategies			
Substance	Top sources	Top pathways *Before treatment* *After treatment*	Top receiving compartments	Suggestions in SFA	Strategies used	Status	Responsibility *EU* *National* *Local*	Pollution movement
Cadmium	1. Goods emissions (car washes largest) 2. Deposition (excluding industries)	Before treatment: 1. Wastewater 2. Stormwater After treatment: 1. Stormwater 2. Wastewater	1. Sediments 2. Sewage sludge (soil not known)	–	Regulation in products, on use, for point-sources and in compartments	Implemented	EU → National	Pipe separation: wastewater/sludge → stormwater
					Cd ban	Ban implemented with exception for artist paint and some batteries	National	
					Certification and upstream work	42 WWTPs Revaq-certified by 2018	Local	
Copper	1. Urban industries, business, services and forestry, and private households	Before treatment: 1. Wastewater 2. Surface runoff After treatment: 1. CSO 1. Wastewater and surface runoff	1. Sewage sludge 2. Surface water	- Focus on non-point emissions - Monitor Cu in urban soils and sediments	Limit values sewage sludge	Implemented	EU → National	Increased efficiency of WWTP treatment: water → sludge CSOs: Receiving water → sludge
					Regulation sludge application on farmland	Implemented	National	
					Regulation industrial discharge	Implemented	National	
					Limit impact of CSO	Water storage constructed	Local	
Pharmaceuticals	1. Households 2. Hospitals	Before treatment: 1. Wastewater 2. CSO After treatment: 1. Wastewater^a^ 2. CSO	Surface water	Treatment: -WWTP (O_3_ or activated carbon) - Hospital (O_3_) - CSO	Upgrade selected WWTPs	10 WWTPs upgraded and 23 planned or under construction	National initiative, local implementation	Potentially: water → sludge

^a^For one of the four compounds studied (ciprofloxacin), the largest share was retained at the WWTP (i.e. either degraded or adsorbed to sludge)

### Comparison of the three substance cases

#### Flows

Households are the largest contributors of both pharmaceuticals and copper, but for the latter, industry is almost equally large. For cadmium, emissions from goods are the largest contributor with emissions from cars via car washes being the largest source.

Wastewater was for all three substances the largest pathway related to the urban water system before treatment. Stormwater was the second largest pathway for cadmium and copper, and CSOs for pharmaceuticals. Wastewater treatment is rather efficient for cadmium and copper and transfers the pollution, to a large extent, to the sludge fraction. The effects of WWTP processes on pharmaceuticals differ between compounds. Some compounds are degraded, transformed or adsorbed to sludge by the conventional treatment processes, while others are largely unaffected (Luo et al. 2014). After treatment, the top pathways for pharmaceuticals had not changed for most of the compounds, while the top pathways were reversed for cadmium, and for copper, CSOs were the largest pathway after wastewater treatment.

Considering that wastewater was the largest pathway of cadmium, one would assume that sewage sludge is the largest compartment, but it is sediments. This is due to run-off over time from historically polluted areas (Bergbäck et al. 2001). For copper, sewage sludge was the largest receiving compartment, followed by surface water. The copper in the surface water ends up in the sediments as well (Bergbäck et al. 2001), but this is not caught by the copper study of Kral et al. (2014) that use different system boundaries with surface water as final compartment and not sediments. Surface water was also the largest receiving compartment for most of the investigated pharmaceuticals.

#### Strategies

Cadmium has long been considered a major environmental issue in many European countries and there are strict regulations in place, both on applications and compartments. The EEC-regulated cadmium concentrations in, e.g. fertilisers (76/116/EEC), and in 1988, an action programme on cadmium were launched with the ambition to limit the use of cadmium through substitution in pigments, stabilisers and plating and recycling of batteries containing cadmium (CEC 1988). Cadmium is also a priority pollutant in the Water Framework Directive^1^ and is considered a substance of very high concern according to REACH (European Chemicals Agency 2013). In Sweden, a ban on the use of cadmium with a few exceptions was introduced already in 1982. It is still used in artist paint and some types of batteries, but there is a particular fee for producing such batteries (Naturvårdsverket 2017).

Unlike cadmium, copper is an essential element, but high concentrations are associated with health risks and environmental damage. In Austria, as well as in most other countries, there are established limit values for copper in different emission ordinances for air, water and soil. Industrial discharges of copper are regulated in the general emission ordinances for wastewater (*Abwasserverordnungen*), which also cover industrial discharges to municipal sewer systems.

Copper and cadmium are both regulated in the EU sludge directive (86/278/EEC). There are further national limit values for the substances in sewage sludge spread on agricultural land. In Austria, only 16% of the sludge was used in agriculture in 2014 (Amann et al. 2017), and in Vienna, all sewage sludge is incinerated (Vanas 2016). In Sweden, 34% of the sewage sludge was spread on agricultural land in 2016 (SCB 2018). In addition to the EU directive and national legislation concerning sewage sludge application on farmland, Sweden has a voluntary certification scheme called Revaq with stricter requirements for sludge quality. By the end of 2018, 42 WWTPs were certified under Revaq, which corresponds to half of the total sludge generation in Sweden (Revaq 2019). To keep the Revaq-certification, the municipal wastewater utilities should successfully carry out ‘upstream work’ (*Uppströmsarbete*), which focuses on controlling and minimising cadmium and other pollutants to the WWTP and improves both sludge quality and effluent.

CSO was identified as an important pathway of copper. In Vienna, combined sewer systems dominate, while separate systems are only found in peripheral parts of the city and cover less than 20% of its area (Stadt Wien 2018). As a response to national legislation introduced in 2007, Vienna has initiated initiatives to limit the impact of CSOs. The most important investment is the *Wientalkanal*, designed to store 110,000 m^3^ of water (Stadt Wien 2018). This initiative is not targeting copper specifically but was introduced to control the pollution related to CSOs in general.

Switzerland is at the forefront of pharmaceutical treatment. In 2016, a law was passed that requires 100 selected WWTPs (out of approximately 700) to be upgraded with ozone or activated carbon until 2040. Large WWTPs (> 80,000 persons), plants serving more than 24,000 persons that also affect drinking water resources and plants for more than 8000 persons that have a small and/or sensitive recipient should be upgraded (Eggen et al. 2014). Ten plants in the country have already been upgraded, and an additional 23 WWTPs are in the process of being upgraded or in the planning phase.^2^

The chosen strategies in these cases may influence pollution movement. For measures of preventive character, there is often no pollution movement. A ban, for example, reduces the total inflow to the system (Eriksson et al. 2011). However, if some compartments are more strictly regulated than others, it may impact pollution movement. For example, in Sweden, cadmium is more controlled in effluent and sludge than in stormwater. A secondary effect of this is that separate pipe systems are increasing, which improves wastewater quality, but at the expense of stormwater quality. Treatment for stormwater can be introduced, which moves cadmium from the water to the sediment compartment (Revitt et al. 2008). However, in Sweden, only 4% of the stormwater is treated in the urban area (Magnusson et al. 2016).

Increasing efficiency of wastewater treatment also leads to pollution movement as an increasing share of the metals are moved from the wastewater to the sludge fraction. When the water in the CSOs is stored as in Vienna and then treated at the WWTP, it may also lead to increased concentrations in the sludge, while reducing concentrations in surface water.

The two methods applied in Switzerland for treatment of pharmaceutical compounds, ozone and activated carbon (granulated (GAC) or powdered (PAC)), function differently. Oxidation often gives rise to transformation products. The transformation products seem to be less toxic than the parent compound, but a polishing treatment step after the ozone treatment to capture transformation products is often recommended (Hollender et al. 2009). If PAC is added to the conventional biological processes, this will lead to more pharmaceuticals in the sludge fraction (Baresel et al. 2017). In Switzerland, sewage sludge is incinerated and will therefore not be spread further in the environment (Chèvre et al. 2013).

### Microplastics in urban water

Since microplastics is a new type of pollution, few abatement and control strategies have yet been implemented. Even if there are no detailed SFAs for microplastics and many sources, pathways and receiving compartments are uncertain, some sources to urban water that likely give rise to important releases have been identified. Similar as for the other substances, households and urban activities, such as road traffic, are considered large contributors (Siegfried et al. 2017). Wastewater, stormwater and CSOs seem to be important pathways to receiving surface waters (Bollmann et al. 2019). In addition to surface water, microplastics are found in large quantities in sewage sludge (Habib et al. 2020) and in stormwater sediments (Borg Olesen et al. 2019). The properties of different microplastics can influence the retention at the WWTP. Microbeads have been shown to be almost completely retained at the WWTP, while fibres are still common in effluent (Sun et al. 2019). Microplastics can also end up in urban soils (Verschoor et al. 2016).

Although there are still many uncertainties regarding the flows of microplastics, many abatement measures and strategies have been suggested. Table 4 shows an overview of suggested strategies and measures for different microplastics sources. For many sources, there are both preventive and decentralised treatment alternatives. Additional treatment at the WWTP has also been explored. This should not be interpreted as an exhaustive list of solutions, but as examples of measures and strategies on different levels. There are probably additional strategies that, depending on the context, may have a large influence on flows of microplastics.

**Table 4:** Overview of sources of microplastics pollution, examples of suggested strategies for the different sources, where in the system it would occur, who will likely have the responsibility if implemented and if implementation will lead to the pollution being moved to another urban compartment. This should not be seen as an exhaustive list of solutions, but as examples of measures and strategies on different levels

Source	Pathway	Suggested measures and strategies	Type *Preventive* *Decentralised* *Centralised*	Responsibility	Pollution movement
Microbeads in PCP’s	Wastewater	Ban/Substitution	Preventive	Producer and authorities for decision and compliance	None
Laundry	Wastewater	Change in practice: textile constructions	Preventive	Producer	None
		Change in washing and consumption behaviour	Preventive	Citizens	None
		Change in practice: pre-washings	Decentralised	Producer and authorities for assuring compliance	Wastewater to solid waste
		Filter in washing machine	Decentralised	Producer and citizens for correct use	Wastewater to solid waste
Plastic pre-production	Wastewater/ stormwater^a^	Enforcement of legislation	Preventive	Authorities	None
		Drain filters	Decentralised	Factories for use and authorities for assuring function	Wastewater to solid waste
Aggregated stormwater		Stormwater retention ponds	Decentralised	Local authorities and/or water and wastewater utilities	Stormwater to sediment
		Street sweepings	Preventive	Local authorities	Stormwater to solid waste
Road traffic	Stormwater^b^	Filter	Decentralised	Road responsible and authorities for assuring compliance	Stormwater to solid waste
		Increase durability and resistance	Preventive	Producer	None
Artificial turfs	Stormwater	Change in maintenance	Preventive	Football field owner and local authorities for assuring compliance	None
		Granulate trap	Decentralised	Football field owner for use and local authorities for assuring compliance	None if returned to field
		Change in material	Preventive	Football field owner	None
Macroplastic	Stormwater	Behavioural change	Preventive	Citizens	Stormwater to solid waste
		Reduce plastic use	Preventive	Citizens	None
		Enhance waste management	Preventive	Waste management	Stormwater to solid waste
Aggregated wastewater		Advanced treatment technologies	Centralised	Water and wastewater utilities	Circulating at WWTP or sludge dependent on technique

^a^Depends on if the spill occurs inside or outside of the facility and if the production plant is connected to a WWTP or not.

^b^Particles that are larger than 10 μm are often deposited close to source, but smaller particles can be transported long distances in air (Kole et al. 2017) and might therefore be deposited in surface water without being transported by stormwater.

#### Suggested strategies to abate microplastic pollution in urban water

An important mitigation measure for microplastics is the ban of microbeads in cosmetic products that have been introduced in many countries (Prata 2018; Xanthos and Walker 2017). However, this ban has received criticism because it only addresses one small source, or even only part of a source as not all cosmetic products are included. It has also been proven difficult to define *plastics* and there have been problems both when excluding and including biodegradable plastics in the ban (McDevitt et al. 2017).

Synthetic fibres are released in connection with washing of synthetic materials (Browne et al. 2011). Such releases in connection with laundry can be reduced by changing the consumption behaviour (i.e. buying textiles that release less fibres) or changing washing habits (Carney Almroth et al. 2018). A decentralised treatment option is a filter in the washing machine (Brodin et al. 2019; Cesa et al. 2020). A similar strategy, but further upstream, is to have the textile manufacturers pre-wash the fabrics. This is already a procedure used in the industry (Carney Almroth et al. 2018). Several studies have found a decrease in fibre release during the initial washes, but there are still substantial releases after the first washes (Cesa et al. 2020; Napper and Thompson 2016). Further, no decrease in fibre release during initial washes has also been reported (Hernandez et al. 2017). Pre-washing will thus not eliminate fibre release from households. Possible preventive actions at the manufacturing level include using and developing knitting techniques that reduce fibre loss and choosing a yarn type that release less fibres (Carney Almroth et al. 2018).

Plastic pre-production plants have been shown to release large amounts of plastic pellets to the surrounding environment (Karlsson et al. 2018). To reduce this, drain filters have been installed in some factories. There is already existing legislation that can be utilised to manage spill during production and transportation, but this legislation needs to be enforced to a larger degree (Karlsson et al. 2018).

There are many sources of microplastics in urban areas, which may end up in stormwater and large quantities of microplastics have been found in stormwater retention pond sediments (Borg Olesen et al. 2019), which indicates that the ponds act as a sink within the urban area. Further upstream, street dust have been shown to contain microplastics, as well as other pollutants (Polukarova et al. 2020) and street sweeping can be used to prevent microplastics from entering the water phase (Vogelsang et al. 2019).

Road traffic is a potentially large source of microplastic pollution in urban areas. A preventive strategy is here to increase the wear resistance of the car tyres. However, the properties for tyres are interrelated. If increasing wear resistance, this will be at the expense of rolling resistance, which is related to fuel consumption, and slip resistance, which is related to safety (Kole et al. 2017). Decentralised treatment techniques, such as filters, has shown a high retention capacity for both microplastics from road traffic and other microplastic particles found in stormwater (Venghaus et al. 2017). However, filter solutions have been criticised by practitioners because installation and the required maintenance may intervene with traffic. Instead filter solutions in stormwater wells are primarily recommended for gas stations and parking lots (Dromberg 2009).

Old car tyres are often used as filling in artificial turfs (Kole et al. 2017). Preventive strategies for this source include substitution to another filling material (Kole et al. 2017) and change in field management. This may include snow disposal and players brushing their clothes before leaving the field (Svenska Fotbollsförbundet 2017). In addition, granulate traps can be used in stormwater wells in the vicinity or in connection with the field drainage system (Svenska Fotbollsförbundet 2017).

Reducing the presence of macroplastics, which break down into microplastics, have been on the agenda longer and there are more strategies for macroplastics in place than for microplastics (Pettipas et al. 2016). For urban water, this particularly concerns littering. A ban, tax or fee on plastic bags can decrease the use and hence the occurrence of litter (Xanthos and Walker 2017). Littering can also be reduced by enhanced waste management and behavioural change campaigns, either voluntary or with economic incentives, such as a deposit-based plastic bottle collection system (Ogunola et al. 2018; Pettipas et al. 2016). The efficiency of behavioural measures can be difficult to assess, but Willis et al. (2018) saw positive effects from such efforts on the amounts of coastal waste.

Additional treatment steps at the WWTP have been suggested as a strategy to reduce the microplastic load to recipient waters (Carney Almroth et al. 2018). The conventional treatments at WWTPs retain much microplastics, but microplastics are still present in effluent (Ngo et al. 2019). Additional treatment steps may further increase the retention capacity. Disc filters, rapid sand filters, dissolved air flotation and membrane bioreactor are treatment technologies that can be used at WWTPs and their efficiency for microplastics has been assessed (Lares et al. 2018; Simon et al. 2019; Talvitie et al. 2017). Most of these technologies were shown to retain a high percentage of microplastics. However, Simon et al. (2019) point out that the necessity to introduce new technologies solely for microplastics needs to be evaluated on a case-by-case basis and compared to other sources in the area. The disc filter tested in Simon et al. (2019) reduced the microplastics load in effluent with an additional 76% in terms of mass, but in total load that only meant a reduction of 3.5 kg/year.

#### Insights for microplastics management in urban water

There are some experiences from the three substance cases that can be important for microplastic management. One aspect is that the main control efforts differed between the substances. For cadmium, the control efforts were mostly preventive, phasing out cadmium from almost all former uses, while the focus for copper has mainly been on limit values in different compartments and discharges. For pharmaceuticals, the main strategy has been treatment far downstream in the system. The difference in strategies between cadmium and copper probably has its background in that copper is an essential element, but toxic in high concentrations, whereas there are no positive effects of cadmium. Cadmium is instead mainly present as an undesirable by-product of essential materials, such as phosphate rock and zinc (van der Voet et al. 1999). The focus on centralised treatment for pharmaceuticals may have two reasons. First, preventive measures, such as bans or substitution, may be difficult as there are ethical issues related to restricting medicine (Eggen et al. 2014). Second, centralised treatment is considered economically preferable if the collection system is already in place and the treatment is expensive (Libralato et al. 2012), which is both true for pharmaceuticals.

For microplastics, most of the suggested strategies and measures are preventive. This can be a consequence of that, like cadmium, microplastics is not essential. However, microplastics arise from many different sources that are often difficult to ban and substitute. Additional treatment at the WWTP was also suggested for microplastics, but there are several differences between pharmaceuticals and microplastics, which may make this option less suitable. Like cadmium and copper, microplastics are largely retained in the conventional WWTP processes and the load of microplastics to recipient waters from WWTPs is mainly a result of large inflows. Further, unlike pharmaceuticals, microplastics seem to have several diffuse sources that do not end up at the WWTP. The experiences from the copper and cadmium cases show that regulations for emissions to stormwater in the urban area are largely absent. This indicates that microplastics in stormwater will be a difficult pathway to manage, as there is a limited structure for control and monitoring.

The sludge fraction is handled differently in the Sweden, Austria and Switzerland. In Sweden, sewage sludge application on farmland is a common application and there has been a large focus on increasing sludge quality. In contrast, the sewage sludge is incinerated in Switzerland and in Austria only a small part is used on farmland. This may give rise to different ways of handling microplastics in wastewater, where initiatives to decrease the loads that reach WWTPs may be perceived as less interesting if the sludge fraction is not used as a fertiliser.

Most of the preventive actions are decided on a societal level, but the practical responsibility tends to fall on two groups: producers and citizens, dependent on where in the system the prevention takes place. Authorities on different levels also have a practical responsibility to control that producers oblige, and that citizens are informed. The mitigation of cadmium also placed some responsibility on producers to substitute or limit the use of cadmium in production processes. Citizens and artist schools have also been targeted with campaigns about cadmium in paint. The Swedish Water and Wastewater Association have been prominent in this work (Svenskt Vatten 2017).

For the advanced centralised treatment technologies, the retained microplastics are often circulating within the WWTP or transferred to the sludge and will be the responsibility of the wastewater utilities. For decentralised treatment, however, the responsibility differs dependent on the source and several actors may have a responsibility. For example, a filter in a washing machine is the responsibility of the producer, but correct use is the responsibility of the citizens. Further, controlling producer compliance is the responsibility of authorities. For most strategies and measures, microplastics are moved from water to solid waste, which makes waste management central. Such movements often mean a shift in responsibility and needs to be coordinated. For coordination and responsibility clarification, a good overview of the system such as can be provided by SFAs is indispensable.

As pointed out by Sedlak (2017), the magnitude of flows and the potential toxicity is not enough for mitigation actions to take place or be successful. If the solution is expensive, there is a need for a significant amount of evidence for actions to be taken. This can be interpreted not just in terms of financial costs, but also costs in terms of impact on the daily life. A ban on microbeads is not a large interference into daily lives, while a ban on synthetic textiles or restrictions on driving is much larger. There is a risk that flows that are difficult to eliminate will be subject to voluntary preventive actions with uncertain impact (Sedlak 2017).

The success of a solution is also related to responsibility allocation. Distinct responsibilities increase the likelihood that a solution will be implemented (Sedlak 2017). For pharmaceuticals, this responsibility has dominatingly fallen far downstream, on WWTPs. For copper, there are several diffuse emission sources in the urban area, such as roofs and brake pads, but these have not been tackled at source. Instead, restrictions have been placed on point emissions upstream (industries) and downstream (sewage sludge). Point sources are both easier to identify and easier to find control measures for than diffuse emissions (Revitt et al. 2013). Stormwater management has been pointed out as an area where unclear responsibilities are a problem (Brown 2005; Wihlborg et al. 2019). Cadmium is a pollutant for which strong regulations have been introduced. Yet, this has not eliminated cadmium in society and management downstream is also required. In practice, it has led to that the end receiver, i.e. wastewater utilities, has the responsibility to handle much of the remaining cadmium in the system. However, wastewater utilities often have limited or no impact over the sources from which cadmium enters the WWTP (Sörme et al. 2003).

There are no established limit values in any compartments for microplastics yet. However, if restrictions are introduced for microplastics in the future, where they are placed will impact management and responsibility. Introduction of strict requirements on WWTP effluents may favour centralised techniques, and the responsibility will fall on wastewater utilities. If microplastics are included in the sewage sludge directive, this may instead of treatment create incentives for source control efforts or increased incineration of sewage sludge. If regulations for microplastics are made part of the water framework directive, this will impact all land-based sources and several urban actors will share the responsibility. These observations are not only valid for microplastics but should be considered by pollutant control management of emerging contaminants in general.

## Conclusion

Microplastics is a new type of pollution in need of efficient and effective management. In this study, we compared flows and strategies to control the flows for three different pollutants to seek guidance for microplastics management. The analysis shows that measures have been introduced on different levels to control the pollution from cadmium, copper and pharmaceuticals, but where in the systems the main control measures have been carried out differ. SFAs can be useful for developing increasingly detailed overviews of sources, pathways and receiving compartments and visualise important and large flows. To gradually develop such overviews has been important to discover and evaluate unknown and neglected sources and interactions. Further, these overviews can highlight parts of the system that needs more attention, such as pharmaceuticals in CSOs, and continuously address administrative and practical responsibility. The microplastic issue is still early in its development and have yet to tackle many of the challenges that belong to the past for cadmium, copper and, to a large extent also for pharmaceuticals. These challenges include developing a more definite overview of sources and pathways and control significant sources. If trusting early assessment for microplastics, sources are numerous, diffuse and most often difficult to control and eliminate. The management of microplastics is further complicated by that microplastics is a diverse pollutant group with different properties. Preventive actions have their advantage in that they most often reduce the amount of microplastics into the system. They are, however, often difficult to implement or need to be combined with other measures to significantly impact the load, especially if they are based on voluntary action. Decentralised treatment requires a distinct allocation of responsibility and the success depends on how the receiving compartment is handled. There is a need for a continuous progress in understanding the movements of microplastics in society, while managing the flows. A systematic approach that combine flow and actor analysis can support management through the development of more and more detailed overviews of the flows of microplastics in urban areas and help highlighting key actors in relation to the flows. It can also facilitate anticipation of how certain regulations of specific flows, compartments or treatment options will impact the whole system and the responsibility for taking different actions.

## Footnotes

1 See Annex II in Directive 2008/105/EC

2 https://www.micropoll.ch/anlagen-projekte/uebersichtskarte/
